# ANCA-associated vasculitis in a HIV-infected patient:a case-based review

**DOI:** 10.1186/s12882-023-03244-9

**Published:** 2023-07-14

**Authors:** Alexandra Vornicu, Bogdan Obrișcă, Bogdan Sorohan, Andreea Berechet, Gener Ismail

**Affiliations:** 1 Department of Nephrology, “Carol Davila” University of Medicine and Pharmacy, Bucharest, 020021 Romania; 2 Department of Nephrology, Fundeni Clinical Institute, Bucharest, 022328 Romania

**Keywords:** ANCA vasculitis, HIV infection, Immunosuppression, Induction therapy

## Abstract

**Background:**

The occurrence of autoantibodies in human immunodeficiency virus (HIV)-infected patients has been previously reported, with a prevalence ranging from 20 to 83%. There are also a few reports of clinically relevant autoantibody profiles in HIV-positive patients that lead to true systemic autoimmune disease; these possible life-threatening diseases have to be considered and treated accordingly.

**Case presentation:**

Here, we present the case of a 29-year-old female patient with a history of well-controlled HIV infection in the last 6 years who was admitted to our department for the evaluation of acute kidney injury and nephrotic syndrome with active urinary sediment. A diagnosis of systemic antineutrophil cytoplasmic antibody (ANCA)-associated vasculitis (AAV) with renal and pulmonary involvement was established. The patient was treated with cyclophosphamide, rituximab and tapering glucocorticoids,and the diffuse alveolar hemorrhage resolved, but the evolution of kidney function was unfavorable, which led to the need to initiate hemodialysis. We highlight the importance of establishing the correct diagnosis, treating the disease accordingly and the possible clinical issues that can appear in a patient with HIV infection during immunosuppressant treatment as induction treatment. Additionally, we performed a thorough literature review of ANCA positivity in HIV-infected patients to properly understand the current evidence.

**Conclusions:**

Although it is not clear whether HIV infection and AAV are causally or coincidentally related, the possibility of this systemic autoimmune phenomenon should be acknowledged by physicians to establish the correct diagnosis and treat the disease accordingly by maintaining a balance between the risks and benefits of immunosuppression in this category of patients, with treatment decisions being made by the members of a multidisciplinary team in centers with experience in AAV.

## Background

Despite remarkable advances in antiretroviral therapy, the prolonged survival of human immunodeficiency virus (HIV)-infected patients has increased their risk of developing kidney disease [1, 2]. Along with HIV-associated nephropathy (HIVAN), a wide spectrum of lesions has been described secondary to intrarenal HIV gene expression or as a consequence of coinfections, comorbid conditions, antiretroviral treatment or immune dysregulation in response to HIV infection [3, 4].

The occurrence of autoantibodies in HIV-infected patients has been previously reported, with a prevalence ranging from 20 to 83% [5, 6]. As reported previously, false-positive results for nonorgan-specific autoantibodies can also be found in healthy people, during infections or in neoplasia [5], but there are also a few reports of clinically relevant autoantibody profiles in HIV-positive patients that lead to true systemic autoimmune disease [7]. Antineutrophil cytoplasmic antibody (ANCA) positivity has been detected with a prevalence between 13 and 83%, without evidence of clinical vasculitis in most cases [6, 8–12]. The incidence of vasculitis in HIV-positive patients is estimated to be less than 1% [7, 13], with some reports of true ANCA-associated vasculitis (AAV) [8, 9, 14–16].

Here, we present the case of a female patient with a history of well-controlled HIV infection who was diagnosed in our department with systemic AAV with renal and pulmonary involvement. We highlight the importance of establishing the correct diagnosis, treating the disease accordingly and the possible clinical issues that can appear during induction immunosuppression therapy in a patient with HIV infection. Additionally, we performed a thorough literature review of ANCA positivity in HIV-infected patients to properly understand the current evidence.

## Case presentation

A 29-year-old female patient without a significant family history was admitted to our nephrology department for the evaluation of acute kidney injury and nephrotic syndrome with active urinary sediment. Her past medical history included a well-controlled sexually transmitted HIV infection in the last 6 years with undetectable viral loads and CD4 counts over 500 cells/mm^3^(939 cells/mm^3^ at admission). She did not have any history of drug abuse, and no opportunistic/concomitant infections were found during the standard screening (tuberculosis was ruled out by performing an Interferon Gamma Release Assay**-** QuantiFERON–TB Gold In-Tube test; hepatitis B virus, hepatitis C virus, CMV, EBV and parvovirus infections were ruled out by specific serology). Her antiretroviral therapy at that time consisted of ritonavir, darunavir and raltegravir. Previously, the renal function of the patient was normal (serum creatinine of 70.7 µmol/L), and no urinary abnormalities or electrolyte disturbances were recorded during the periodical follow-ups in the Infectious Disease Unit. There were six weeks between the first signs of renal involvement and admission to our nephrology department.

At admission, her physical examination revealed a normal body temperature, an altered general status, pitting edema, a blood pressure of 150/70 mmHg and a heart rate of 92/minute. Auscultation revealed regular heartbeats and diminished breath sounds in the lung bases with an oxygen saturation of 92% on room air. She reported hemoptysis in the past 2 weeks before admission. Abdominal palpation did not reveal any hepatomegaly, splenomegaly or signs of ascites. Her urine output was 1000 ml/day.

The initial laboratory evaluation showed an increased serum creatinine of 503.8 µmol/L and urea of 45.78 mmol/L;metabolic acidosis, with a serum bicarbonate of 12.4 mmol/l;and serum sodium and potassium within normal ranges. Urinalysis was positive for red blood cells (127 cells/µl) and proteins; 24-hour proteinuria was 7 g with a serum albumin of 2.4 g/dl. Anemia was diagnosed, with a hemoglobin of 6.2 g/dl and low erythrocyte indices; no thrombocytopenia was recorded during follow-up. The mean cellular volume was 68.4 fL, the serum iron was 101 µg/dL, the ferritin was 899 ng/ml and the transferrin was 156 mg/dl. White blood cell counts were in the normal ranges.

Immunological screening was performed and revealed positivity for ANCA (titer of anti-myeloperoxidase antibody (anti-MPO) of 52.4 IU/ml), and a low complement C3 fraction(67 mg/dl). Tests for anti-GBM antibodies were negative, and the value of C-reactive protein was elevated (10 µg/mL).

**Fig. 1A:**
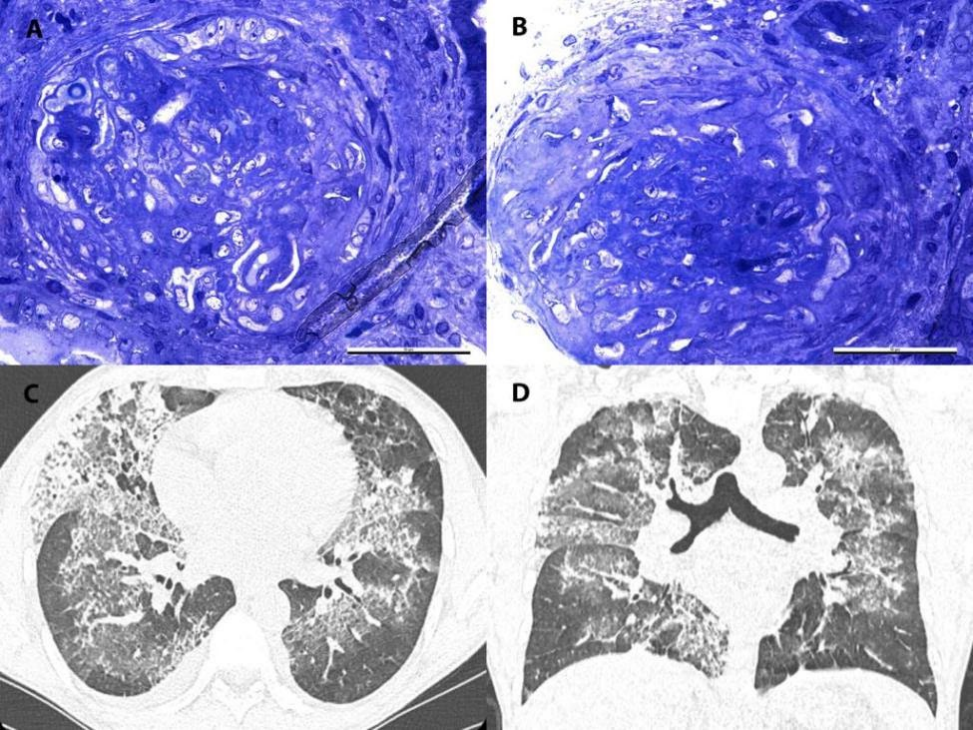
, **1B-Kidney biopsy**: Light microscopy (toluidine blue staining)- Functional glomeruli with fibrocellular crescents. **Figure 1C, 1D-Computed tomography**: Alveolar infiltrates suggestive of diffuse alveolar hemorrhage

A percutaneous ultrasound-guided kidney biopsy was performed. The immunofluorescence was pauci-immune. Under light microscopy, there were 8 glomeruli for examination,of which 5 were globally sclerosed. The functional glomeruli presented fibrocellular crescents (Fig. 1A and B) and fibrinoid necrosis. There was mild interstitial fibrosis and tubular atrophy. There were also signs of acute tubular injury with a simplification of the tubular epithelium with intraluminal cellular detritus and a tubulointerstitial mononuclear infiltrate. Electron microscopy showed diffuse foot process effacement and no dense deposits.

Additionally, computed tomography was performed and revealed alveolar infiltrates suggestive of diffuse alveolar hemorrhage (Fig. 1C and D).

**Fig. 2:**
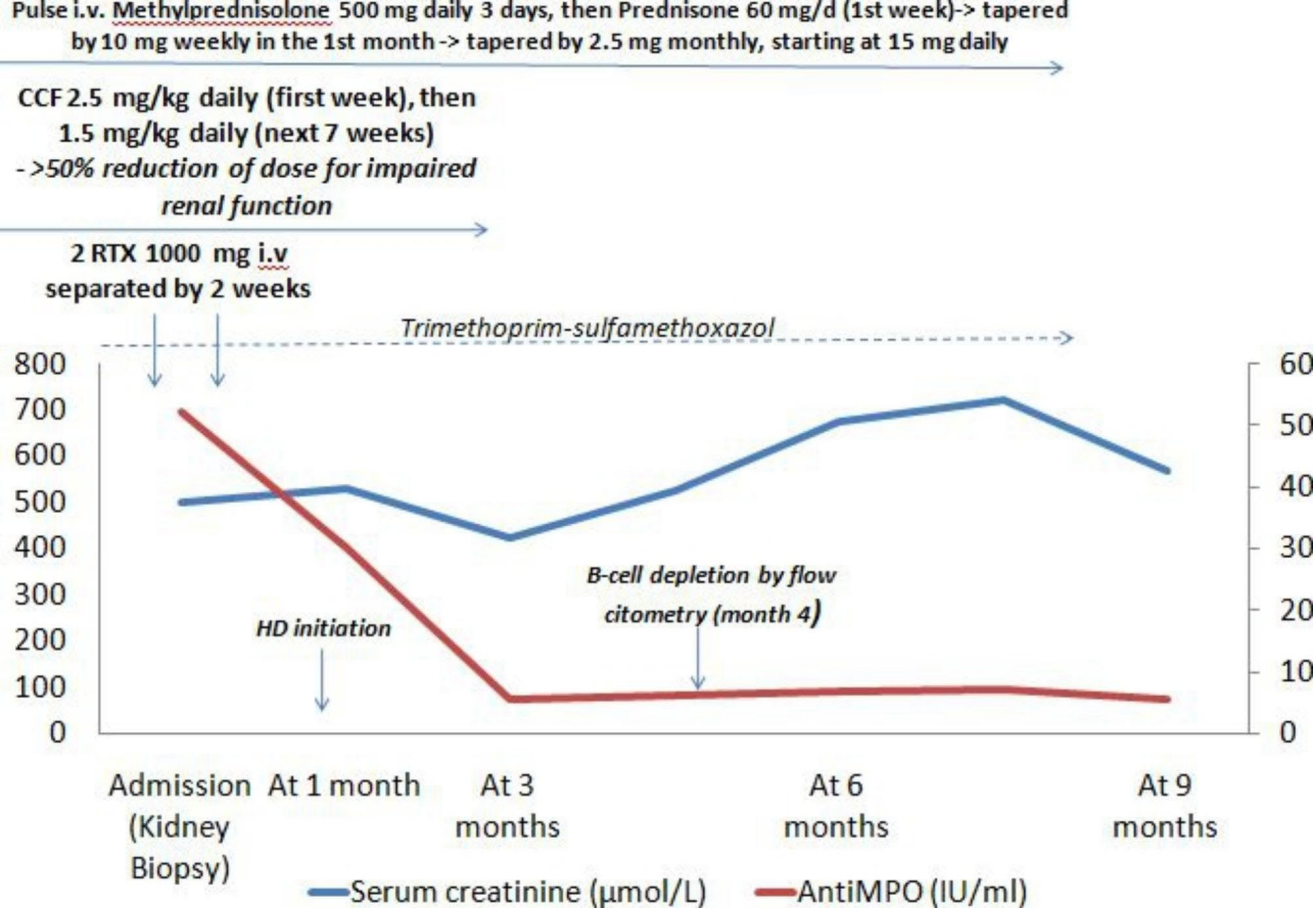
Timeline of the patient’s disease course

A diagnosis of systemic AAV with renal and pulmonary involvement was established, with a Birmingham Vasculitis Activity Score (BVAS) of 18 points. The patient was started on a remission induction immunosuppressive treatment regimen consisting of a combination of cyclophosphamide, rituximab and tapering corticosteroids,which led to resolution of the diffuse alveolar hemorrhage and immunological remission, but owing to renal function loss,hemodialysis was initiated (Fig. 2). The patient received prophylaxis for Pneumocystis pneumonia with trimethoprim-sulfamethoxazole, and no infectious complications were reported during immunosuppression treatment.

## Discussion and conclusions

Despite the improvement in survival after the introduction of antiretroviral therapy, HIV-infected patients have an increased risk of developing kidney disease [1, 2]. Along with HIV-associated nephropathy, a wide spectrum of lesions has been described that could involve each compartment of the kidney, the mechanisms involved being a direct association with HIV infection, the consequences of superinfections and antiretroviral treatment and the systemic immune response to HIV infection [3, 4]. Furthermore, the spectrum of HIV-related kidney disease has changed with the widespread use of effective antiretroviral therapy, with conditions such as nephrotoxicity of antiretroviral therapy, focal segmental glomerulosclerosis not otherwise specified, arterionephrosclerosis of aging and hypertension, diabetic nephropathy and immune complex-mediated glomerulonephritis having an increased prevalence in this population of patients in recent years [1, 2]. Here, we report one case of true AAV with renal and pulmonary involvement in a young patient with a history of well-controlled HIV infection.

The dysregulation of the B and T lymphocyte interaction, molecular mimicry, viral triggers (such as Epstein‒Barr virus (EBV)), cytokine production (such as tumor necrosis factor-α (TNFα)) and polyclonal activation of B lymphocytes may favor the development of autoantibodies in patients with HIV infection [5, 7–9, 12, 15, 17], and infections usually have a critical role in the breakdown of tolerance and the appearance of autoreactivity [12]. As reported previously, false-positive results for nonorgan-specific autoantibodies can also be found in healthy people, during infections or in neoplasia [5]; in HIV-infected patients, several studies have reported the occurrence of autoantibodies with a prevalence ranging from 20 to 83% [5, 6], with few reports of clinicallyrelevant autoantibody profiles that lead to true systemic autoimmune disease [7].

Most of the studies were conducted in the preantiretroviral therapy era with a population of patients presenting at a more advanced disease stage, but in the last ten years, systematic investigations have started to appear with the purpose of describing the prevalence of autoantibodies in the antiretroviral therapy era when a higher proportion of patients have a good immunovirological status [5, 7, 18]. Iordache et al. found an ANCA positivity rate of 13% in 92 HIV1-infected patients with a good immunovirological status [5] compared to historical studies where the prevalence was up to 83% [6, 10, 12], suggesting that viral replication may play a pivotal role in the development of autoantibodies in HIV infection. The study conducted by Meng supports this observation by demonstrating that antiretroviral therapy suppresses autoimmune manifestations by reducing CD33 + CD11b + HLA-DR + myeloid-derived suppressor cells in vivo [18].

Another contrasting hypothesis is that autoimmune diseases and HIV infection are coincidental pathologies and that autoimmune phenomena are possibly being modified in the context of immune deficiency secondary to HIV infection; in this situation, highly active antiretroviral therapy (HAART) might hasten the onset of autoimmune diseases [7]. Additionally, some autoimmune diseases,such as vasculitis, sarcoidosis and thyroid disease, may also represent a form of immune reconstitution inflammatory syndrome secondary to the initiation of HAART [7].

According to the hypothesis proposed by Zandman-Goddard and Yehuda Shoenfeld in 2002 [19], there are four stages of autoimmune manifestations in HIV-infected patients based on the CD4 count and the viral load:for autoimmunity to appear, the patients have to have a preserved immune system, as in acute HIV infection, when even if the viral load is high, the CD4 count is still high (stage I);during the cellular response when the CD4 count is normal/low and the viral load is high (stage II); and in stage IV when the CD4 count is high and viral load is low secondary to HAART. During immune deficiency with a CD4 count less than 200 cells/mm^3^ and a high viral load, autoimmune diseases are not found based on this classification, with the exception of those diseases that are driven by CD8 + T lymphocytes [19]. We can classify our patient in stage IV using the classification of Zandman-Goddard and Yehuda Shoenfeld, taking into account that she was under stable antiretroviral treatment in the past six years with undetectable viral loads and CD4 + T lymphocyte counts over 500 cells/mm^3^. We believe that in this particular situation, autoimmunity has been favored by the restoration of immune competence with functional T-cell reconstitution in a patient with probably altered immune regulation and/or genetic susceptibility.

The incidence of vasculitis in HIV-positive patients is estimated to be less than 1% [7, 13]. In 1992, Gherardi in 1992 reported the findings of their study of 148 patients with symptomatic HIV infection in whom muscle, nerve or skin biopsies were performed. Thirty-four of the patients (23%) presented inflammatory vascular diseases, with 30 patients presenting exclusively small vessel involvement; there was no specification of ANCA status [17]. In this case report, small vessel vasculitis was found with cutaneous and neurologic involvement in more than half of the patients [17]. Another case of ANCA-negative granulomatous necrotizing vasculitis was reported by Garcia-Garcia et al. [20] based on muscle biopsy; the patient had poorly controlled HIV and was diagnosed in an advanced stage with a CD4 count of 175 cells/µl and a viral load of 120,000 copies/ml. The treatment was based on cyclophosphamide, vincristine, adriamicin, corticosteroids and the initiation of antiretroviral therapy [20].

A retrospective study conducted by Peraldi [21] included 92 HIV-positive patients with an episode of acute kidney injury, 60 of whom had renal biopsies performed. No small vessel vasculitis was diagnosed in this subgroup of patients with HIV infection, acute kidney injury and a poor immunovirological status, as 82% of the patients had overt AIDS, and the mean CD4 cell count was 76/mm^3^. 80% of the patients were on at least one antiretroviral agent, with only 13% being on a three antiretroviral agent protocol [21]. Vali et al. [22] reported the results of the biopsy sample analyses of 27 HIV-infected patients with renal dysfunction, and almost half of them had nephrotic-range proteinuria (48% of the patients). The CD4 count of the patients ranged between 77 and 633/µl, and 29.6% of the patients were on HAART. The authors reported only one case of pauci-immune crescentic glomerulonephritis, without mentioning the ANCA status of the patients [22].

In a review conducted by Reville in 2000 [23] with the aim of describing HIV-associated rheumatic disease reported in the literature, the authors also presented their department experience. Of the 458 patients referred to rheumatology consultation over 6 years, only 4 patients (1%) were diagnosed with vasculitis: 1 case of Wegener’s granulomatosis, 1 case of polyarteritis nodosa, 1 case of central nervous system angiitis and 1 case of cutaneous vasculitis [23]. Another recent review focused on autoimmune disease in HIV-positive patients was published in 2014 by Iordache et al. [7];these authors also reported their retrospective experience consisting of 52 patients with autoimmune diseases and HIV infection. There were 11 cases of vasculitis (1 case of cutaneous vasculitis and 1 case of granulomatosis with polyangiitis), and in 9 of these cases, the patient had a CD4 level > 200 mm^3^. Seven of the patients needed immunosuppression, and the treatment consisted of corticosteroids in 7 patients, cyclophosphamide in 1 patient, intravenous immunoglobulins in 1 patient, methotrexate in 2 patients and plasmapheresis in 1 patient; HAART was started in 9 patients. The patients’ conditions showed a good evolution, as 8 of the patients achieved complete remission [7].

The results of the literature review of reported study cohorts/case reports of patients with ANCA positivity are presented in Table 1. ANCA positivity has been detected with a prevalence between 13 and 83%, without evidence of clinical vasculitis in most cases [6, 8–12]. Some studies reported no correlation between ANCA positivity and opportunistic infection [11], hypergammaglobulinemia [5, 11, 16] or the stage of the disease [6, 11]. On the other hand, some studies have found an apparentcorrelation between ANCA positivity and disease stage [5, 16, 24] (Table 1). In a cross-sectional observational study conducted by Iordache et al. [5],the authors found an inverse association between the presence of more than one autoantibody and the CD4 + lymphocyte count (p 0.03) [5].

There are some reports of true AAV [8, 9, 14–16]. The reported manifestations in HIV-positive patients with ANCA-associated vasculitis were renal [8, 9, 14, 16], pulmonary [9, 15, 23], articular [8, 16], cutaneous [16], ophthalmological [8] and neurological [14]. The renal manifestations reported included acute kidney injury [8, 9] and nephritic syndrome [8, 9, 14] (Table 1). In our patient, a diagnosis of systemic AAV with severe renal and pulmonary involvement was established; the patient presented with acute kidney injury and nephrotic syndrome with active urinary sediment, and renal biopsy sample analysis and computer tomography confirmed the diagnosis in the context of ANCA positivity.

The treatments reported in patients with HIVand AAV was based on glucocorticosteroids [8, 9, 14–16, 23], cyclophosphamide [9, 15], rituximab [8] and the initiation of HAART [9, 15], and some of the patients had a well-controlled HIV infection [8], while others showed ANCA positivity at the time of HIV infection diagnosis with a low CD4 count [9, 15, 25]. Additionally, there were two reports of Churg Strauss vasculitis in HIV-infected patients [26, 27] with renal involvement in the case reported by Myugen (nephritic syndrome) [26]; in both cases,vasculitis was diagnosed simultaneously with HIV infection. In both cases, the treatment was based on corticosteroids [26, 27]. Our patient, who had a well-controlled HIV infection with an undetectable viral load and a CD4 count over 500 cells/mm^3^, was started on an induction immunosuppressive treatment regimen consisting of a combination of cyclophosphamide, rituximab and tapering corticosteroids,which led to the resolution of the diffuse alveolar hemorrhage and immunological remission, but owing to renal function loss,dialysis was initiated. In this case, correct antibiotic prophylaxis prevented infectious complications despite the patient being under aggressive combined immunosuppressive therapy.

According to the aforementioned studies in Table 1, it was not the isolated presence of ANCA positivity in HIV-infected patients that guided the treatment but the clinical manifestations (renal and pulmonary in particular). In all situations when there were clinical manifestations of ANCA vasculitis, the immunosuppressant treatment included glucocorticosteroids.

**Table 1:** Cases of ANCA Positivity in HIV-Infected Individuals Reported in the Literature

Study	Type of study	Number of patients	Immunovirological status	ANCA positivity	Clinical manifestations	Correlation with the stage of the disease	Treatment
Evans [8]	Case report	1	CD4 > 400 cells/mm^3^ UndetectableVL (under Ritonavir and Darunavir)	pANCA > 100 IU/ml	Renal (Acute kidney injury, nephritic syndrome), articular, ophthalmological	No	Glucocorticosteroids, rituximab
Romano [25]	Case report	1	CD4 27 cells/mm^3^ VL-NR Untreated before	cANCA 251–736 UA/ml	No clinical vasculitis	At diagnosis	-
Mirsaedi [9]	Case report	1	CD4 146 cells/mm^3^ VL- 91,469 c/ml Untreated before	pANCA 68 U/ml	Renal (Acute kidney injury, nephritic syndrome), pulmonary	At diagnosis	Glucocorticosteroids, cyclophosphamide, HAART
Mohapatra [15]	Case report	1	CD4 0.9/µl Viral load- NR Untreated before	cANCA- titer NR	Pulmonary	At diagnosis	Glucocorticosteroids, Cyclophosphamide, HAART
Jansen [10]	Case report	1	CD4 18 × 10^6^/l Untreated before	cANCA 1/320	No clinical vasculitis	At diagnosis	HAART
Davenport [14]	Case report	1 patient	CDC grade 4 disease -at diagnosis	Perinuclear with some focal intracytoplasmic positivity (IF)	Renal (intermittent hematuria and proteinuria) and neurologic (peripheral neuropathy)	-	Corticosteroids
Reville [23]	Review (1 case report)	1 patient	NR	cANCA positivity titer NR	Severe sinusitis and pulmonary involvement (infiltrates)	NR	Corticosteroids
Klassen [16]	Case report Cross-sectional	-82 pt. +: -1 index pt. presented separately -1 pt. presented separately	VL-not reported Treatment- NR − 21 asymptomatic HIV+ (CD4 > = 0.5 × 10^9^/l -26 asymptomatic HIV+(CD4 < 0.5 × 10^9^/l -26) -10 pt. with ARC (CDC group IV A) -14 pt. with AIDS-OI -11 pt. with AIDS- MAL	-5 ANCA+/10 ARC -2 ANCA+/14 AIDS-OI -4 ANCA+/11-AIDS-MAL -No ANCA positivity in asymptomatic HIV; pANCA-3 pt. cANCA- 8 pt	Index patient-articular, cutaneous 1 Dutch pt.- renal involvement 48 pt. -No clinical vasculitis	Correlation with disease stage- “The occurrence of ANCA was limited to the symptomatic stages of HIV infection with the exception of the index pt.”	Corticosteroids
Cornelly [11]	Observational Prospective	199	Median CD4 80/ul VL-NR 77%-AIDS 76% on ART	20% ANCA + (IF) Atypical ANCA 67%, pANCA 33%	No clinical vasculitis	No correlation with the stage of the disease.	-
Sorrentino [24]	Observational	88	VL, CD4-NR Treatment-NR (49-asymptomatic HIV; 39- symptomatic HIV)	ANCA positivity: 21/39 (53.8%)-symptomatic HIV 2/49 (4.1%)- asymptomatic HIV	No clinical vasculitis	Correlation with disease stage	-
Iordache [5]	Cross-sectional	92 pt.	HAART 85% Median mean CD4 611/mm3 VL undetectable in 74% of pt.	At least 1 Ab (45%) ANCA-13%	12% from the whole cohort had > = 1 clinically relevant Ab	Ab presence is associated with CD4 count.	-
Savige [12]	Observational	105 pt.	55 pt. were treated with Zidovudine and 34 were not. CD4 counts < 400/ul in 78 pt.; >400/ul in 11 pt. Asymptomatic infection- 37 pt. ARC − 32 pt. AIDS-36 pt.	ANCA+-18 pt. cANCA-4 pANCA-4 atypical-10	No clinical vasculitis	There was no significant correlation between ANCA positivity and immunological status.	-
Koderisch [6]	Observational	29 patients (45 sera)	VL-NR Median T4/T8 ratio 0.43 3-asymptomatic 16- lymphadenopathy syndrome 5- ARC 5-AIDS (2-KAPOSI)	31 sera/24 patients- faint homogenous cytoplasmatic staining of neutrophils (IF) 4 sera/3 patients-faint cANCA 0-pANCA; 9sera/5 pt.-borderline + ANCA-ELISA 3 pt- mpo-ELISA +	No clinical vasculitis	No correlation between ANCA and stage of disease.	-
Iordache [7]	Retrospective descriptive	52 patients -> 1 case of granulomatosis with polyangiitis	Good immunovirological status	Transiently cANCA +	GPA confirmed histological	-	Immunosuppressant treatment (the drugs NR)

AIDS- acquired immunodeficiency syndrome, AIDS-OI- AIDS and opportunistic infections; AIDS-MAL- AIDS and secondary malignancies, ARC-AIDS-related complex; HIV-human immunodeficiency virus, Ab-antibody,VL-viral load, pANCA-perinuclear anti-neutrophil cytoplasmic antibody,cANCA- cytoplasmic anti-neutrophil cytoplasmic antibody, NR-not reported, ART- antiretroviral therapy, HAART-highly active antiretroviral therapy; IF-immunofluorescence; pt-patients; GPA-granulomatosis with polyangiitis; ELISA- enzyme-linked immunosorbent assay

The KDIGO 2021 Clinical Practice Guideline for the Management of Glomerular Diseases [28] recommends the administration of glucocorticoids in combination with cyclophosphamide or rituximab for the initial treatment of new-onset AAV; inpatients presenting with a markedly reduced or rapidly declining glomerular filtration rate (serum creatinine > 4 mg/dl), a combination of rituximab and cyclophosphamide can also be considered [28–30], as we have also tried in our patient whose case is reported here. The KDIGO 2021 Clinical Practice Guideline for the Management of Glomerular Diseases [27] also states that plasma exchange can be considered in patients with a serum creatinine > 5.7 mg/dl or in those with alveolar hemorrhage with hypoxemia, although the PEXIVAS trial showed no benefit with the addition of plasma exchange regarding the incidence of death or end-stage kidney disease in patients with AAV presenting with GFR < 50 ml/min/1.73 m^2^ or alveolar hemorrhage [31].

For HIV-related kidney disease, there is no consensus regarding immunosuppressant treatment in addition to antiretroviral therapy [28]. Immunosuppressive drugs can favor the development of opportunistic infections in HIV-positive patients [29], and as there are no controlled clinical trials, each case needs to be discussed by the members ofa multidisciplinary team; corticosteroids should be administered in patients with potentially life-threatening vasculitis, and cytotoxic agents should be tried with caution in resistant cases [23, 32]. In their case-based review of 52 HIV patients with autoimmune disease, Iordache and coauthors concluded that positivity for HIV should not limit the use of immunosuppressive therapy, as this was well tolerated by these patients [7].

In conclusion, although it is not clear whether HIV infection and AAV are causally or coincidentally related, the possibility of this systemic autoimmune phenomenon should be acknowledged by physicians to establish the correct diagnosis and treat the disease accordingly by maintaining a balance between the risks and benefits of immunosuppression in this category of patients, with the treatment decisions being made by the members of a multidisciplinary team in centers with experience in AAV.

## Data Availability

The datasets used during the current study are available from the corresponding author on reasonable request.

## List of Abbreviations

AAV: ANCA-associated vasculitis
Ab: Antibody
AIDS: Acquired immunodeficiency syndrome
AIDS-MAL: AIDS and secondary malignancies
AIDS-OI: AIDS and opportunistic infections
ANCA: Antineutrophil cytoplasmic antibody
Anti-MPO: Anti-myeloperoxidase antibody
ARC-AIDS: Related complex
ART: Antiretroviral therapy
BVAS: Birmingham Vasculitis Activity Score
cANCA: Cytoplasmic anti-neutrophil cytoplasmic antibody
EBV-Epstein: Barrvirus
ELISA: Enzyme-linked immunosorbent assay
GPA: Granulomatosis with polyangiitis
HAART: Highly active antiretroviral therapy
HIV: Human immunodeficiency virus
HIVAN: HIV-associated nephropathy
IF: Immunofluorescence
NR: Not reported
pANCA: Perinuclear antineutrophil cytoplasmic antibody
Pt: Patients
TNFα: Tumor necrosis factor-α
VL: Viral load
